# Spacing Analysis of Casting Dolly Windows for Tunnel Sidewall Lining Based on the Flow Characteristics of Freshly Mixed Concrete

**DOI:** 10.3390/ma17081800

**Published:** 2024-04-14

**Authors:** Zuoqiang Chi, Shuai Shao, Yimin Wu, Shuai Yang, Zhuangzhuang Zhou

**Affiliations:** 1 Shandong Luqiao Group Co., Ltd., Jinan 250014, China; 13505318812@163.com; 2 School of Civil Engineering, Central South University, Changsha 410075, China; wuyimin531@csu.edu.cn (Y.W.); 224811051@csu.edu.cn (S.Y.); 3 China State Construction International Investments Limited, Nanjing 211100, China; zzz15162122893@163.com

**Keywords:** tunnel engineering, sidewall pouring, fresh concrete, flow characteristics, analysis of dolly window spacing

## Abstract

During the actual construction of tunnel sidewall lining, construction workers often use only one or two windows per layer for pouring in order to reduce the construction sequence, which often leads to a reduction in the quality of tunnel sidewall concrete pouring. Therefore, this study analysed the necessity of the window-by-window pouring of sidewall lining through the study of concrete flow characteristics of the tunnel sidewall lining pouring process, and the reasonable spacing of pouring windows was analysed. This study firstly verified the accuracy of the simulation parameters and the feasibility of the simulation method of the lining pouring process through indoor experiments and simulation analyses, and then it numerically simulated and analysed the flow of concrete during the lining pouring process of tunnel sidewalls. The following conclusions were made: the smaller the slump of the freshly mixed concrete, the higher the pumping flow rate; additionally, the shorter the one-time pouring distance, the smaller the spacing of the trolley feeding window should be. Furthermore, this study makes suggestions for the reasonable spacing of pouring trolleys under several working conditions.

## 1. Introduction

Concrete is one of the most widely used construction materials in modern times, and it is an essential raw material for tunnel construction [1]. Tunnel lining formed by the pouring of concrete guarantees the safe operation of the whole tunnel. However, tunnel lining, which is put into operation after pouring, still has a lot of quality problems, for example, blank lining, which causes a loss of structural strength and durability problems [1], and cracks, which cause water seepage and leakage problems [2]; these problems are detrimental to the safe operation of tunnels [3]. One of the reasons for these quality problems is the low quality of the lining concrete.

A large number of scholars have conducted research to improve the quality of tunnel lining. Yoshitake et al. [4] developed a measurement system for tunnel lining surfaces, which allows for tunnel lining quality to be quantified during inspection. Harseno et al. [5] used ground-penetrating radar to detect the blank lining of tunnel lining and improved the method, which is able to estimate the thickness of blank lining. Judit Gómez et al. [6] applied a distributed fibre optic sensor system to an underpass tunnel and verified the reliability of the system for structural health inspection. Rosso et al. [7] developed a method to indirectly measure tunnel disease, which demonstrated an improved measurement efficiency. Gao et al. [8] proposed a new method for tunnel disease detection: this method repeatedly examines the same point of the lining at different times, thus improving the accuracy of the detection. Other scholars have also attempted to improve the quality of tunnel lining. Larive, Catherine et al. [9] proposed the idea of mixing fibres in concrete, which enhances the ductility and flexural strength of concrete; enhances the waterproofing properties of concrete; and, at the same time, reduces the thickness of lining concrete. Iskhakov, Tagir et al. [10] enhanced the deformability of concrete by incorporating soft inclusions and air bubbles into it, and this effectively improved the ageing and cracking of tunnel lining. Yun, Kyong Ku et al. [11] replaced silica fume with colloidal silica as a mineral admixture in concrete, which improved the strength, durability, and pumpability of the concrete and reduced the possibility of concrete cracking. Wang et al. [12] investigated the role of steel-fibre-reinforced concrete in tunnel lining and showed that steel-fibre-reinforced concrete helps to reduce the pressure on the lining and maintain the stability of the supporting structure.

In a large number of studies, the concrete admixture has been altered to improve its performance; however, there is a scarcity of studies attempting to improve the quality of lining based on the actual engineering situation. Starting with the flow characteristics of the concrete itself and based on the actual pouring situation in the field, our research team reveals the flow characteristics of concrete in the lining formwork through a numerical simulation, with the aims of analysing the flow characteristics of concrete in the pouring process and determining the reasonable spacing of the pouring windows of tunnel lining trolleys under certain working conditions so as to provide suggestions for the pouring scheme of concrete in various projects.

## 2. Feasibility Verification of Simulation Scheme and Acquisition of Rheological Parameters

In this step, the rheometer test, slump extensibility test, and L-box flow test were carried out, and an analysis of the concrete flow test was carried out by using a CFD Eulerian multiphase flow simulation. By comparing the results of the tests, the feasibility of the simulation scheme was verified while providing the rheological parameters that meet the requirements of field casting for simulation in the subsequent sections.

### 2.1. Concrete Flow Test

#### 2.1.1. Test Raw Materials

The raw materials used in this study included cement, fine aggregate, coarse aggregate, fly ash, a water-reducing agent, and water. The details of the raw materials are the same as those in the literature [13] published by our team (the team is affiliated with the School of Civil Engineering, Central South University, China).

#### 2.1.2. Test Conditions

A total of six different concrete compositions were selected for testing: M1 was the concrete proportion scheme for the actual second lining casting on site; M2, M3, and M1 had the same sand ratio but a different water–cement ratio; and M4, M5, M6, and M1 had the same water–cement ratio but a different sand ratio. These six compositions were selected to verify the feasibility of the simulation scheme, and their details are shown in Table 1.

#### 2.1.3. Brief Description of Tests and Results

1. Rheometer test

A TR-CRI concrete rotational rheometer, produced in Shanghai, China, was selected for the rheometer test, and the specific details are the same as those in [13]. The steps of the test were as follows:

(1) Freshly mixed concrete was loaded into the cylinder of the TR-CRI rheometer, and the initial shear rate was set to 0.85 rps.

(2) A cross-shaped rotor was inserted into the concrete sample to a depth of about 100 mm. Time–torque variation (T-N) curves of the concrete at speeds of 0.80 rps, 0.75 rps, 0.7 rps, 0.65 rps, 0.6 rps, 0.55 rps, 0.5 rps, 0.45 rps, 0.4 rps, and 0.35 rps were measured in decreasing order to obtain the torque values of the concrete at each speed.

(3) Similarly, a cruciform rotor was inserted into the concrete sample at a depth of about 200 mm, and the torque value of the concrete was tested at each rotational speed.

(4) The torque values of the concrete measured before and after the implementation of the same rotational speeds were subtracted to obtain 10 average torque values, and the software on the rheometer automatically imported these 10 sets of torque values and fitted them to a 150 mm rotor height to determine the yield stress and plastic viscosity of the concrete.

(5) The rheological parameters of each group of specimens were recorded.

The obtained test results are shown below (Table 2).

2. Slump extension test

For the slump extension test setup, the most common collapsibility cylinder in China was adopted; the specific details of the test setup and steps are shown in [13]. When the concrete no longer slumped or the whole slumping time reached 30 s, the distance between the highest point of the specimen and the height of the cylinder was measured with a ruler and used as the slump value of the concrete specimen. For the extensibility test, the process of lifting off the slump cylinder must be completed within 5–10 s. After the concrete stopped flowing, the maximum diameter of the unfolded circle was measured, as well as the diameter in the direction perpendicular to the maximum diameter, and the average of the two was taken as the mean value of the degree of expansion. Three parallel tests were carried out for each concrete composition, and the average values were taken. The specific test results are shown in Table 3.

3. L-box flow test

In the L-box flow test, the traditional L-box test model was used, and three parallel tests were conducted and averaged for each group of conditions, the details of which can be found in [13]. In this test, the time of concrete flow to each characteristic point (T200, T300 … T700) was recorded; the time was accurate to 0.1 s, and the whole test process was completed within 5 min. The specific results are shown in Table 4.

### 2.2. Selection and Accuracy Verification of Rheological Parameters

In this section, a numerical simulation is presented, which serves to determine the rheological properties of freshly mixed concrete at the macroscopic level. Freshly mixed concrete is mainly regarded as a fluid composed of mortar and dense particles in the discrete phase. However, the three major flow laws of fluid, namely, the law of the conservation of mass, the law of the conservation of momentum, and the law of the conservation of energy, are all described by a system of nonlinear equations, which makes it difficult to obtain an analytical solution with the methods traditionally used to solve the problem. Computational fluid dynamics (CFD), based on theoretical equations of fluid dynamics, provides both theoretical and practical support in solving complex flow problems, and it has become an important research tool after theoretical analytical and experimental methods [14]. In this section, a CFD Eulerian multiphase flow simulation is used to simulate and analyse the concrete flow test, and the accuracy of the rheological parameters and the feasibility of the simulation scheme are verified by comparing with the test results in Section 2.1.

#### 2.2.1. Numerical Simulation of Slump Tests

**Figure 1:**
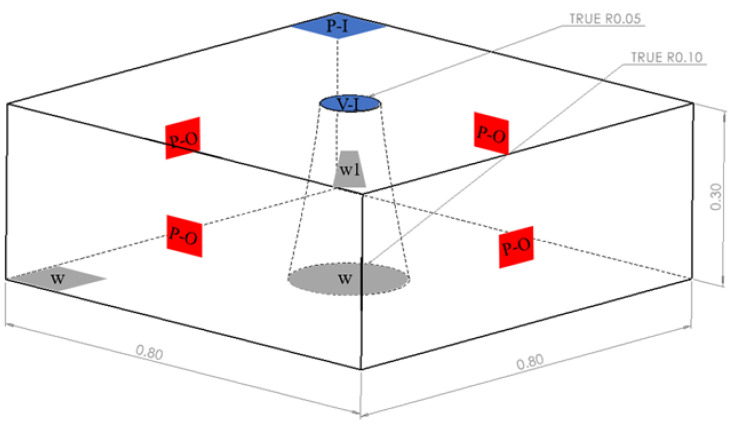
Slump test modelling diagram (V-I, velocity-inlet; P-I, pressure-inlet; P-O, pressure-outlet; Both w and w1 are wall).

In order to accurately simulate the flow of concrete during the test above, this study used SOLIDWORKS 2016 software(Version: SOLISOLIDWORKS 2016, Dassault Systemes, Massachusetts, USA), establishing the model shown in Figure 1 below (the upper surface of the conical table has a radius of 0.05 m, the lower bottom surface has a radius of 0.1 m, and the height is 0.3 m) as the initial filling area of the concrete, while the remaining parameters of the model, as well as the boundary conditions, were set up as shown in [7].

Regarding the six sets of slump tests that were carried out, the particle diameter in the simulation was 5 mm, the volume fraction of the particles within the concrete was 0.4, the filling time of the concrete was 30 s, and the flow rate of the mortar was 0.023 m/s.

The final simulation results are shown in Table 5.

A comparison was made, and it was found that the test value was slightly smaller than the numerical solution, but this was reasonable. In the slump test, the wall of the cylinder was not completely smooth, and the concrete was disturbed during the lifting of the slump cylinder; the numerical simulation process did not take into account the fact that the slump cylinder disturbed the concrete, which caused the abovementioned deviation. These results are the same as those obtained in [13].

In addition to the reasons mentioned above, the most important sources of variability were the computational characteristics of the Eulerian multiphase flow model itself: due to the high fluidity of concrete, the Eulerian method had limitations in treating solid particles as the basic assumption for the proposed fluid treatment, and complex particle size distributions could not be represented in the simulation; in addition, the Eulerian method did not take the size of the particles into account in the simulation, which also contributed to the bias in the results of the simulation [15].

#### 2.2.2. Numerical Simulation of L-Box Tests

**Figure 2:**
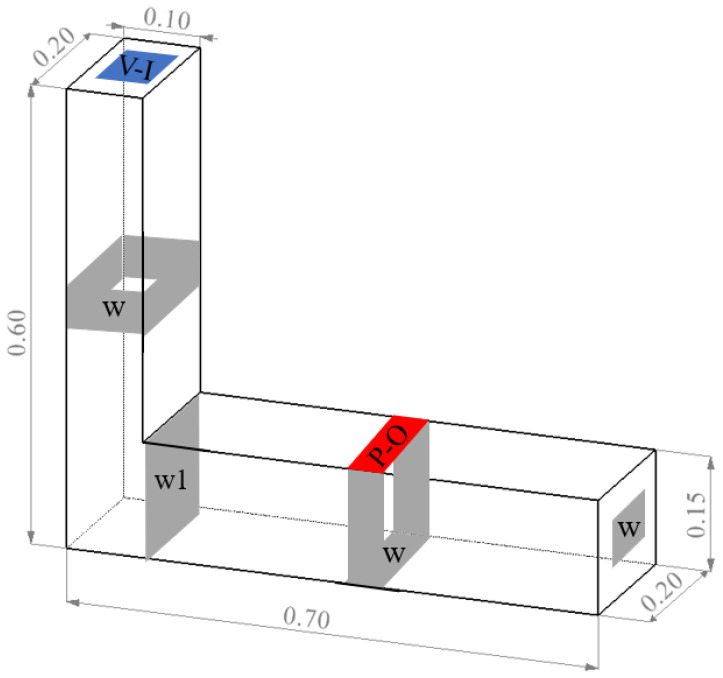
L-box test model dimensions and boundary settings (V-I, velocity-inlet; P-O, pressure-outlet; Both w and w1 are wall).

As with the slump simulation test, in order to accurately simulate the flow of concrete into the L-shaped box during the test, the authors used SOLIDWORKS 2016 software to build the L-shaped space shown in Figure 2 below (the length × width × height of the left vertical cubic column was 0.2 m × 0.1 m × 0.6 m, and the length × width × height of the right transverse cubic column was 0.7 m × 0.2 m × 0.15 m). The vertical and horizontal cubic columns were the initial filling area and flow area of the concrete, respectively.

In order to minimise the influence of the size of the divided grid cells on the accuracy of the calculation results, the grid was divided into 240,000 cells, and the quality of the grid was considered 1. The rest of the steps were generally similar to those of the slump simulation. The simulation results are shown in Table 6.

A comparison of the results showed that the experimental values were generally higher than the numerical solutions, with the numerical simulation providing better flowability of the concrete. This was because the spacer movable door was not smooth, and the numerical simulation similarly ignored the problem of the perturbation of the initial state of the concrete by the lifting of the spacer movable door and assumed that the spacer movable door was removed straight away. Furthermore, as previously mentioned, the main sources of error were the computational properties of the Eulerian model itself.

Through an analysis of the above test values and numerical solution comparison results, it was found that the corresponding errors were within a reasonable range; therefore, the use of CFD Eulerian multiphase flow simulation technology to simulate the concrete flow scheme was feasible, and the values obtained from the rotational rheometer test could be selected for the subsequent simulation of the rheological parameters. Thus, in this study, the rheological parameters of the M3, M4, and M5 conditions were selected as the simulation parameters, as are detailed in Table 7.

## 3. Simulation and Characterisation of Concrete Flow under Unpressurised Feeding Conditions in Sidewalls

For the actual lining structures of railway and road tunnels, generally, a large amount of whole-mould lining concrete is poured; thus, commercially available software (including ANSYS FLUENT) often requires huge computational resources in the calculation of the fluid, resulting in great difficulties in implementing full-sized, three-dimensional pouring simulations. In order to ensure the smooth progress of the simulation, this study simplified the simulation scheme as follows:

(1) A more realistic two-dimensional simulation scheme was chosen;

(2) The effect of attached vibrators and inserted vibrators on the enhancement of concrete flow properties was ignored when performing the simulations;

(3) Only the effect of reinforcement bars perpendicular to the concrete flow direction in the lining of the concrete was considered.

### 3.1. Model Introduction and Boundary Condition Setting

**Figure 3:**
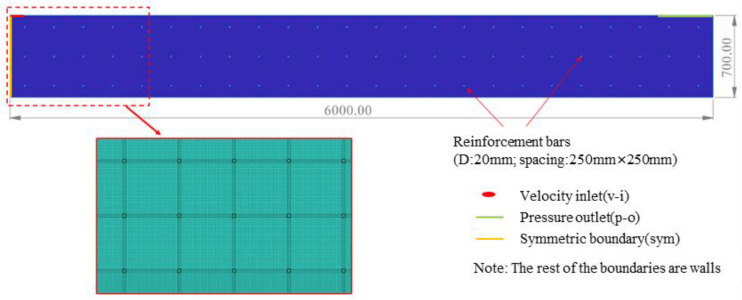
Tunnel sidewall lining casting model with grid.

“Technical Specifications for Construction of Highway Tunnel” (JTG/T 3660-2020), “Technical Guidelines for Railway Tunnel Engineering Construction” (TZ 204-2008), “Technical Specification for Construction of High Speed Railway Tunnel Engineering” (Q/CR 9604-2015), and other specifications stipulate that the difference in the height of the concrete before and after moving the trolley should be less than 0.5 m during the lining pouring process. For this reason, the authors developed a pouring model (6 m long and 0.7 m high), as shown in Figure 3 below, and divided it into 22,0716 grid cells to ensure calculation accuracy. According to actual pouring conditions, the left boundary was set as the symmetrical boundary (sym) to simulate the flow characteristics of the concrete in a 12 m long range; the upper left was set as the velocity inlet, with the width of the inlet set to 0.2 m and the size of the inlet converted from the actual pumping flow rate of each working condition; the upper right was set as the pressure outlet, with a pressure value of 1 standard atmospheric pressure; and the remaining boundaries were set as non-slip walls.

### 3.2. Selection of Working Conditions for Calculation

The flow characteristics of concrete in tunnel lining sidewalls are mainly affected by the flow characteristics of the concrete itself and the pumping speed. According to the technical tunnel construction and concrete pumping specification requirements, three groups of concrete with slumps of 184 mm, 199 mm, and 219 mm, measured in the previous test, were selected to analyse the longitudinal flow characteristics of concrete in sidewalls under different slumps (working conditions 1, 2, and 3). Then, according to actual casting requirements, three pumping flow rates of 40 m^3^/h, 50 m^3^/h, and 60 m^3^/h under a 199 mm caving degree were selected to analyse the longitudinal flow characteristics of concrete in sidewalls under different pumping flow rates (working conditions 2, 4, and 5). The specific working conditions are shown in Table 8.

### 3.3. Longitudinal Flow Characteristics of Concrete at Different Slumps

**Figure 4:**
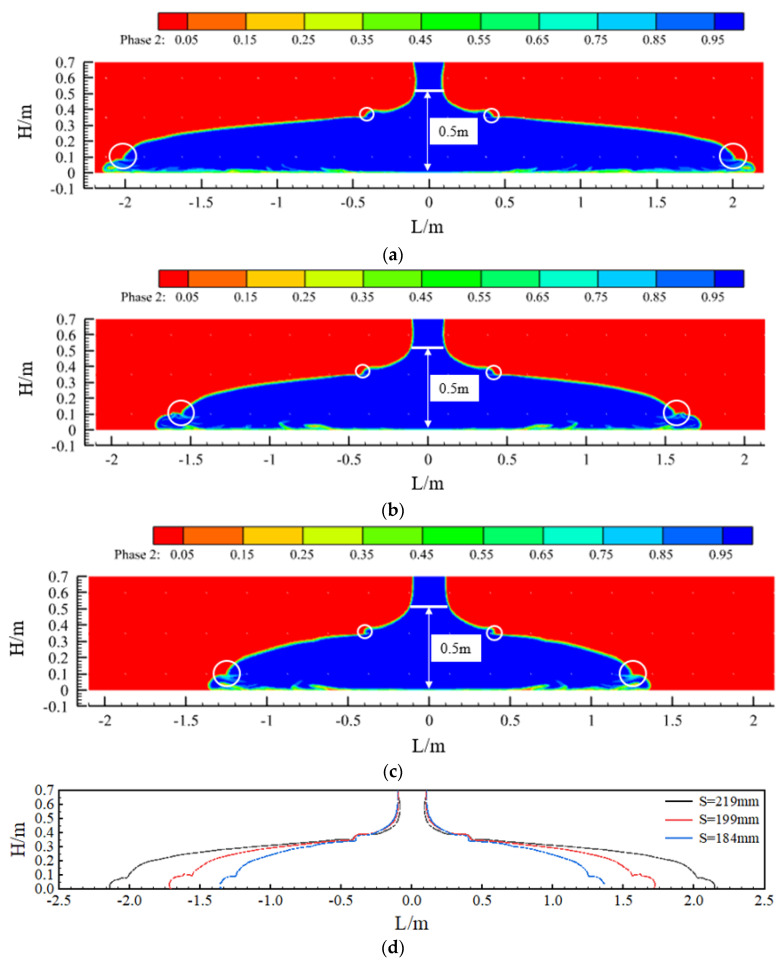
The effect of concrete casting under different slumps and comparative diagrams: (**a**) slump S = 219 mm; (**b**) slump S = 199 mm; (**c**) slump S = 184 mm; and (**d**) comparison chart.

Figure 4 shows the effect of concrete placement and a comparison of the concrete in the sidewalls at three slumps (S = 219 mm, 199 mm, and 184 mm). When the concrete was pumped at a flow rate of 50 m^3^/h, the concrete flowed to both sides at a specific initial speed under the action of its own weight, and the flow characteristics of the concrete differed greatly with the different slumps. First, due to the blocking effect of the steel bars in the formwork, when the concrete flow contacted the first and second layers of steel bars, a leap phenomenon occurred on both liquid surfaces. The points of the two jumps were close, they became closer to the pouring point as the slump decreased, and the jumps weakened as the pouring continued. Second, comparing the three sets of calculated conditions, the slope of the concrete liquid level curve between the two leaping points increased with a decrease in the concrete slump. Finally, under the fulfilment of the specification requirements, from the point of view of the final pouring distance, when the slump of the concrete was 219 mm, 199 mm, or 184 mm, the final flow distance of the concrete was 4.3 m, 3.4 m, or 2.7 m, respectively. Thus, the final pouring distance became increasingly shorter with a decrease in the slump of the concrete.

**Figure 5:**
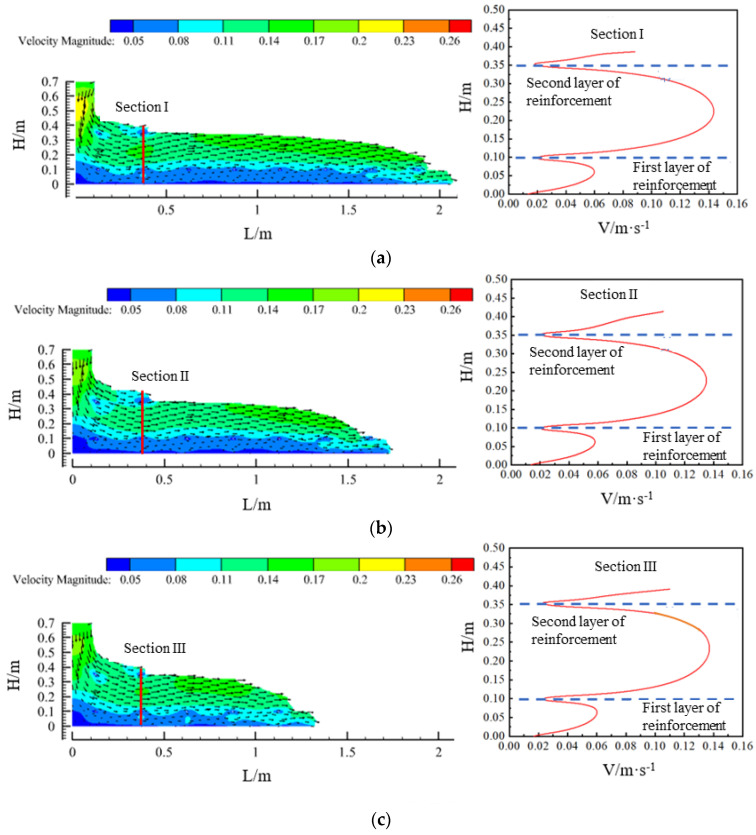
Velocity vector plots of the flow field at each slump and cross-section velocity profiles: (**a**) slump S = 219 mm; (**b**) slump S = 199 mm; and (**c**) slump S = 184 mm.

Velocity vector plots of the flow field in the concrete region within the model for the three slumps are shown in Figure 5. The concrete was blocked by the lowest layer of reinforcement, as well as the viscous resistance of the formwork, thus showing an overall stratified flow. The planes (sections I, II, and III) were intercepted at 0.375 m horizontally, and, by analysing the change in the velocity of the concrete after passing through the first and second layers of reinforcement, it could be seen that the flow velocity of the concrete between the first and second layers of reinforcement showed a trend of first increasing and then decreasing, and the flow velocity of the concrete between the first layer of reinforcement and the base plate also showed the same trend. The maximum flow velocity between the two layers of reinforcement was about 0.14 m/s, and there was little difference in the flow velocity of the concrete in the flow field at each slump.

### 3.4. Longitudinal Flow Characteristics of Concrete with Different Pumping Flow Rates

**Figure 6:**
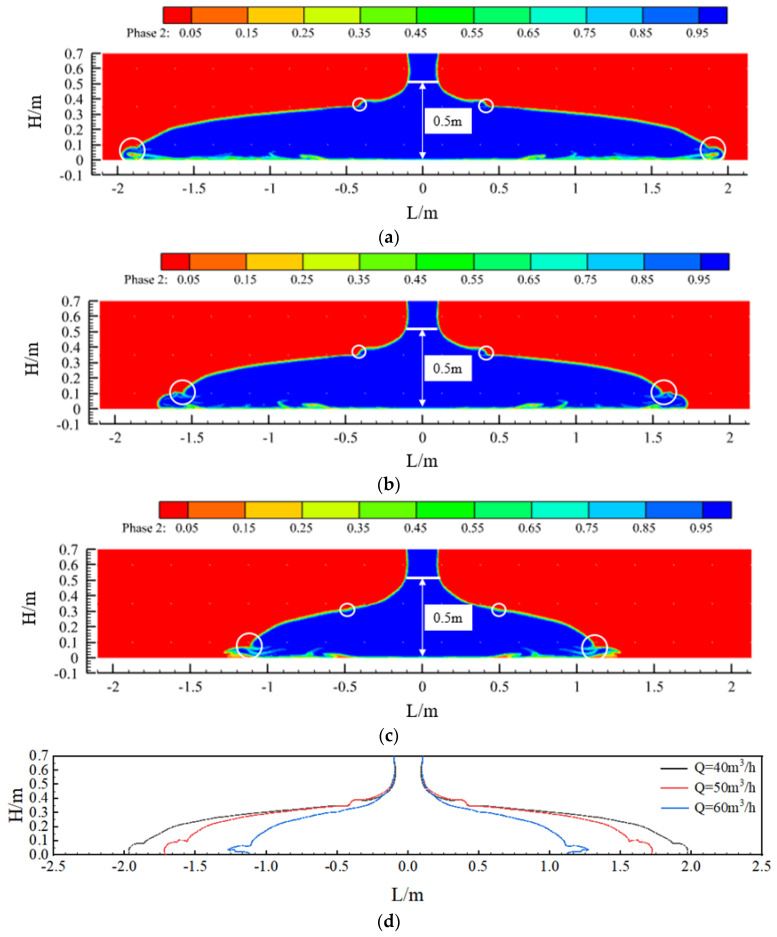
Pouring effect of concrete at different pumping flow rates and comparison charts: (**a**) pumping flow Q = 40 m^3^/h; (**b**) pumping flow Q = 50 m^3^/h; (**c**) pumping flow Q = 60 m^3^/h; and (**d**) comparison chart.

Figure 6 shows the pouring effect and a comparison of the concrete in the sidewall at three pumping flow rates (Q = 40 m^3^/h, 50 m^3^/h, and 60 m^3^/h). Due to the blocking effect of the reinforcement in the formwork, the liquid level of the concrete in the formwork showed two jumps. The flow characteristics of the concrete varied with the different pumping flow rates; the higher the pumping rate, the more obvious the effect of vertical concrete build-up in the formwork, and the slope of the concrete level curve became increasingly larger. When the concrete pumping speed was increased from 40 m^3^/h to 50 m^3^/h and then to 60 m^3^/h, the maximum pouring distance of the concrete also reduced from 3.9 m to 3.4 m and, finally, to 2.5 m while meeting the requirements of the specification.

**Figure 7:**
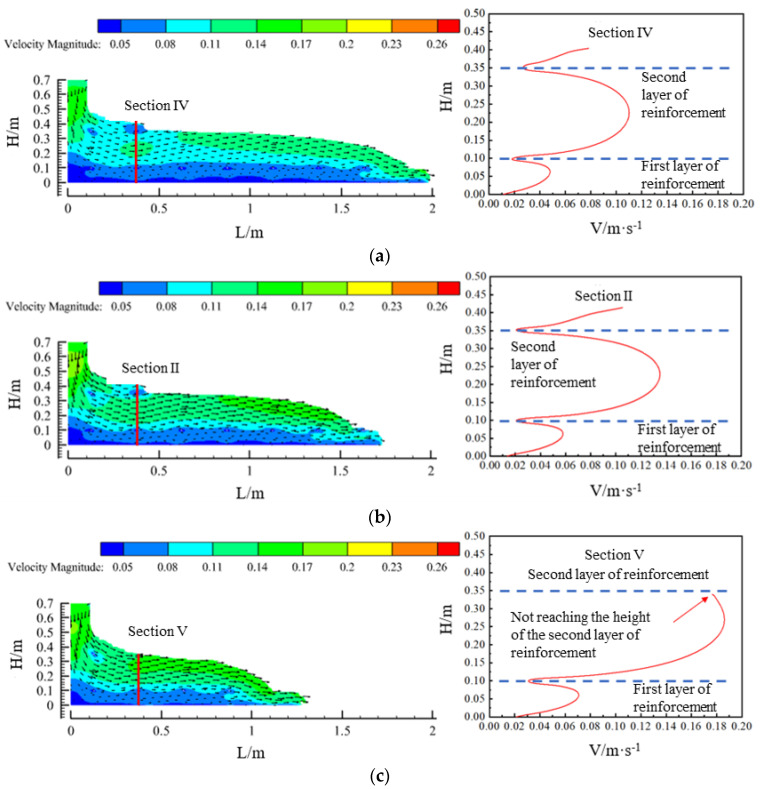
Vector plot of flow field velocity at each pumping flow rate and cross-section velocity profile: (**a**) pumping flow Q = 40 m^3^/h; (**b**) pumping flow Q = 50 m^3^/h; and (**c**) pumping flow Q = 60 m^3^/h.

Velocity vectors of the flow field in the concrete region of the model for the three pumping flow rates are shown in Figure 7. The concrete showed a stratified flow phenomenon due to the obstruction of the lowest reinforcement and the friction of the formwork. With the increase in the pumping flow rate, the overall flow rate of the concrete in the flow field also increased. When analysing the intercepted planes (sections II, IV, and V) and the concrete between the first and second layers of reinforcement, it was found that the flow rate showed a trend of increasing and then decreasing, and the concrete flow pattern in the middle of the first layer of the reinforcement and the bottom slab showed the same trend. Additionally, as the concrete pumping flow rate was increased from 40 m^3^/h to 50 m^3^/h and then to 60 m^3^/h, the maximum flow rate of the concrete between the two layers of reinforcing steel increased from 0.11 m/s to 0.14 m/s and, finally, reached 0.18 m/s.

## 4. Analysis of Dolly Window Spacing for Sidewall Lining Pouring

At present, in engineering practice, two common problems occur in the pouring process of tunnel lining sidewalls. Firstly, according to the corresponding tunnel design and construction specifications, the tunnel lining sidewalls must be poured symmetrically window by window, while on-site workers usually use only one or two windows per layer for pouring in order to reduce the construction sequence [16]. Secondly, the spacing of the windows of the existing tunnel lining casting trolley is relatively fixed [17], but it can be seen through the above simulation that the spacing of the maximum windows required under different slumps and pumping flow rates is different, and it is likely that the conflict between the two leads to a reduction in the quality of concrete casting. For this reason, this subsection focuses on the characteristics of the longitudinal transport of concrete sidewalls under three slumps (Cases 1, 2, and 3), and it proposes a reasonable pouring scheme to analyse the reasonable spacing of windows while analysing the necessity of the window-by-window pouring of sidewalls.

In the simulation in Section 3.3, it could be seen that, when the slump of the concrete was 219 mm, the maximum unilateral flow distance of the concrete within the specification requirements was 2.15 m. Therefore, in the first pouring window, when the difference between the height of the concrete level before the start of the pour and after the completion of the pour reaches 0.5 m, the pour is stopped; then, the pouring of concrete starts from the window of 4.3 m (twice the maximum flow distance on one side), which ensures that the concrete poured in the two windows will connect. If the spacing between the casting windows is too large, the quality of the casting in the articulation area may be poor, which is the reason for the window-by-window casting of the sidewalls mentioned above. Additionally, if the spacing between the casting windows is too small, a large number of casting windows are required, which is wasteful and not conducive to improving the efficiency of the construction.

**Figure 8:**
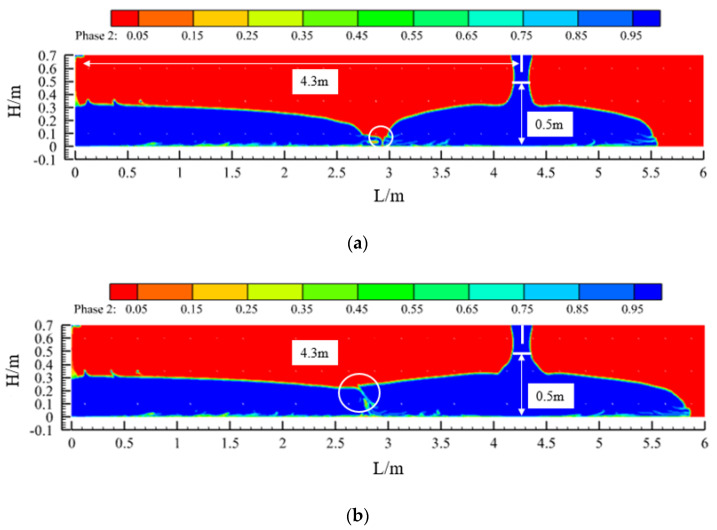
Pouring programme design I (Case 1: slump S = 219 mm): (**a**) concrete contact between two window pourings and (**b**) during the pouring of the second window.

Figure 8 shows the flow state of the concrete before and after the process, i.e., when it just makes contact and after a period of pouring. After the concrete has been in contact for a period of time, the slope of the liquid level on one side of the contact is relatively smooth compared to that on the other side, and the difference in the liquid level of the concrete between before and after the process is less than 0.5 m, which is in accordance with the requirements of the specification. Similarly, when the slump of the concrete is 199 mm or 184 mm, the pouring of concrete from the window of 3.4 m or 2.7 m, respectively, also complies with the specification.

**Figure 9:**
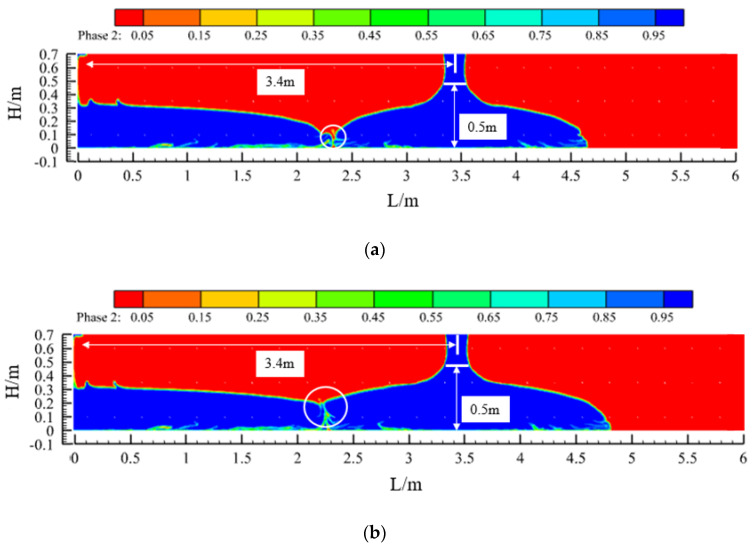
Pouring programme design II (Case 2: slump S = 199 mm): (**a**) concrete contact between two window pourings and (**b**) during the pouring of the second window.

**Figure 10:**
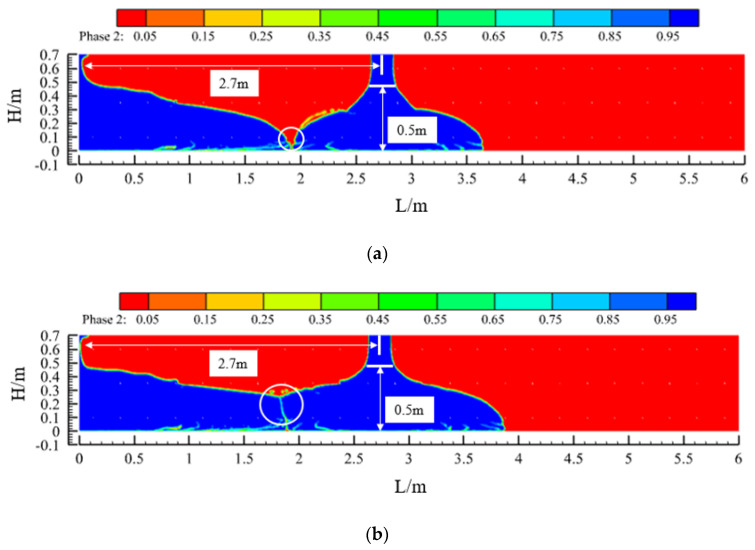
Pouring programme design III (Case 3: slump S = 184 mm): (**a**) concrete contact between two window pourings and (**b**) during the pouring of the second window.

In addition, in Figure 8, Figure 9 and Figure 10, the contact point of the concrete in each pouring process can be seen (the circled part in the picture). Due to the presence of air and other factors, the concrete poured before and after the contact process will form a contact surface, and the contact surface tends to slump toward the concrete side poured first. The quality of the concrete poured on the contact surface is lower, which is one of the factors in the formation of cold joints.

The above simulation shows that, when the slump of the concrete is 219 mm, 199 mm, or 184 mm, the reasonable spacing of the trolley feed windows can be determined based on Table 9 so as to improve the quality of concrete pouring.

## 5. Conclusions

This study has focused on the simulation and characterisation of concrete flow under unpressurised feeding conditions during the casting of tunnel sidewalls, and the following conclusions are made:

1. During the pouring process of the sidewall, the freshly mixed concrete flowed from the pouring window to the two sides at a specific initial speed under the action of its own gravity, and then the concrete was blocked by the reinforcement in the formwork. When the concrete flowed into contact with the first two layers of reinforcing bars, the liquid level exhibited a leap phenomenon. With a decrease in the concrete slump and an increase in the pumping flow rate, the slope of the concrete level curve between the two leaping points showed an increasing trend, and the leaping phenomenon continued to weaken as the pouring continued.
2. During the sidewall pouring process, the freshly mixed concrete showed an overall velocity stratification phenomenon, and the upper concrete flow velocity was larger. The slumping degree in the flow field had little effect on the flow velocity of the concrete, while, with an increase in the pumping flow rate, the overall flow velocity of the concrete in the flow field increased significantly.
3. Under the requirement of meeting the specifications, regarding the final pouring distance, the smaller the slump of the concrete and the larger the pumping flow rate, the smaller the distance of one-time pouring; additionally, the larger the pumping flow rate, the more obvious the effect of the vertical stacking of the concrete in the formwork. Therefore, the smaller the slump of freshly mixed concrete and the larger the pumping flow rate, the smaller the spacing design of the trolley feed windows should be. In this study, on the basis of the analysis, design values of the window spacing of trolley casting have been suggested for several working conditions, as are shown in Table 9.

## Figures and Tables

**Table 1:** Concrete mix ratio of each concrete composition.

Grouping Number	Cement	Admixture	Fine Aggregate	Coarse Aggregate 1	Coarse Aggregate 2	Additives	Water	Water–Cement Ratio	Sand Ratio	Water-Reducing Agent Dosage
M1	291	125	749	323	749	4.16	178	0.43	0.41	1%
M2	287	123	749	323	749	4.16	184	0.45	0.41	1%
M3	283	122	749	323	749	4.16	189	0.47	0.41	1%
M4	291	125	710	334	777	4.16	178	0.43	0.39	1%
M5	291	125	783	312	726	4.16	178	0.43	0.43	1%
M6	291	125	819	301	701	4.16	178	0.43	0.45	1%

**Table 2:** Test results of concrete rheological parameters for each concrete composition.

Grouping Number	$\mathrm{Yield}\;\mathrm{Stress}\;\tau_{0}$ (Pa)	$\mathrm{Plastic}\;\mathrm{Viscosity}\;\mu_{p}$ (Pa·s)
M1	140	136
M2	188	127
M3	126	107
M4	544	176
M5	312	225
M6	476	266

**Table 3:** Test results of slump extension of each concrete composition.

Grouping Number	Mean Value of Slump S (mm)	$\mathrm{Mean}\;\mathrm{Value}\;\mathrm{of}\;\mathrm{Extension}\;D_{f}$ (mm)
M1	201	503
M2	208	515
M3	219	529
M4	184	491
M5	199	517
M6	203	505

**Table 4:** Concrete L-box flow test results of different concrete compositions.

Grouping Number	T_200_ (s)	T_300_ (s)	T_400_ (s)	T_500_ (s)	T_600_ (s)	T_700_ (s)
M1	2.5	3.9	5.3	6.0	8.2	11.5
M2	2.2	3.1	4.3	5.8	8.1	11.4
M3	1.8	2.7	3.8	5.0	6.1	7.3
M4	3.3	5.1	8.7	13.9	21.5	30.4
M5	2.6	4.5	6.8	10.6	16.1	22.3
M6	2.3	4.6	7.8	12.3	18.5	27.9

**Table 5:** Slump extensibility test results and numerical results.

Test Condition	Slump (mm)			Extension (mm)		
	Test Value	Numerical Solution	Error (%)	Test Value	Numerical Solution	Error (%)
M1	201	218	8.5	503	523	4.0
M2	208	223	7.2	515	522	1.4
M3	219	229	4.6	529	524	0.9
M4	184	199	8.2	491	521	6.1
M5	199	208	4.5	517	537	3.9
M6	203	205	1.0	505	518	2.6

**Table 6:** L-box test results and numerical results.

Condition	Projects	T_200_ (s)	T_300_ (s)	T_400_ (s)	T_500_ (s)	T_600_ (s)	T_700_ (s)
M1	Test Value	2.5	3.9	5.3	6.0	8.2	11.5
	Numerical Solution	0.6	1.3	2.4	3.5	5.3	9.4
M2	Test Value	2.2	3.1	4.3	5.8	8.1	11.4
	Numerical Solution	0.6	1.3	2.3	3.5	5.6	10.3
M3	Test Value	1.8	2.7	3.8	5.0	6.1	7.3
	Numerical Solution	0.5	1.1	1.9	2.9	4.3	7.6
M4	Test Value	3.3	5.1	8.7	13.9	21.5	30.4
	Numerical Solution	0.9	2.1	3.7	7.1	13.8	24.2
M5	Test Value	2.6	4.5	6.8	10.6	16.1	22.3
	Numerical Solution	0.9	2.0	3.3	5.6	10.3	18.0
M6	Test Value	2.3	4.6	7.8	12.3	18.5	27.9
	Numerical Solution	1.1	2.4	4.2	7.4	13.6	23.2

**Table 7:** Values of sidewall concrete parameters for numerical simulation.

Grouping Number	Yield Stress (Pa)	$\mathrm{Plastic}\;\mathrm{Viscosity}\mu_{p}$ (Pa·s)	Slump (mm)	Extension (mm)
M3	126	107	219	529
M4	544	176	184	491
M5	312	225	199	517

**Table 8:** Simulated working conditions for the lining of sidewalls.

Case	$\mathrm{Yield}\;\mathrm{Stress}\;\tau_{0}$ (Pa)	$\mathrm{Plastic}\;\mathrm{Viscosity}\;\mu_{p}$ (Pa·s)	Slump (mm)	Pumping Flow (m^3^/h)	Flow Rate (m/s)
Case 1	126	107	219	50	0.154
Case 2	312	225	199	50	0.154
Case 3	544	176	184	50	0.154
Case 4	312	225	199	40	0.123
Case 5	312	225	199	60	0.185

**Table 9:** Spacing of trolley windows at different slumps.

Case	Yield Stress (Pa)	$\mathrm{Plastic}\;\mathrm{Viscosity}\;\mu_{p}$ (Pa·s)	Slump (mm)	Window Spacing (m)
Case 1	126	107	219	4.3
Case 2	312	225	199	3.4
Case 3	544	176	184	2.7

## Data Availability

All data are available from the authors.

## References

[1] Zamora-Castro S.A., Salgado-Estrada R., Sandoval-Herazo L.C., Melendez-Armenta R.A., Manzano-Huerta E., Yelmi-Carrillo E., Herrera-May A.L. (2021). Sustainable Development of Concrete through Aggregates and Innovative Materials: A Review. Appl. Sci..

[2] Lee S.-J., Kee S.-H. (2023). Effect of Thickness of an Air Cavity behind Concrete Tunnel Linings on 400 MHz GPR Time Signals: Numerical Simulation and Analysis. JKSNT.

[3] Protopapadakis E., Voulodimos A., Doulamis A., Doulamis N., Stathaki T. (2019). Automatic Crack Detection for Tunnel Inspection Using Deep Learning and Heuristic Image Post-Processing. Appl. Intell..

[4] Yoshitake I., Maeda T., Hieda M. (2018). Image Analysis for the Detection and Quantification of Concrete Bugholes in a Tunnel Lining. Case Stud. Constr. Mater..

[5] Harseno R.W., Lee S.-J., Kee S.-H., Kim S. (2022). Evaluation of Air-Cavities behind Concrete Tunnel Linings Using GPR Measurements. Remote Sens..

[6] Gómez J., Casas J.R., Villalba S. (2020). Structural Health Monitoring with Distributed Optical Fiber Sensors of Tunnel Lining Affected by Nearby Construction Activity. Autom. Constr..

[7] Rosso M.M., Marasco G., Aiello S., Aloisio A., Chiaia B., Marano G.C. (2023). Convolutional Networks and Transformers for Intelligent Road Tunnel Investigations. Comput. Struct..

[8] Gao X., Yang Y., Xu Z., Gan Z. (2024). A New Method for Repeated Localization and Matching of Tunnel Lining Defects. Eng. Appl. Artif. Intell..

[9] Larive C., Bouteille S., Berthoz N., Zappelli S. (2020). Fiber-Reinforced Sprayed Concrete as a Permanent Tunnel Lining. Struct. Eng. Int..

[10] Iskhakov T., Timothy J.J., Plückelmann S., Breitenbücher R., Meschke G. (2023). Compressible Cementitious Composite Materials: Multiscale Modeling and Experimental Investigation. Cem. Concr. Compos..

[11] Yun K.K., Kim J.B., Song C.S., Hossain M.S., Han S. (2022). Rheological Behavior of High-Performance Shotcrete Mixtures Containing Colloidal Silica and Silica Fume Using the Bingham Model. Materials.

[12] Wang X., Fan F., Lai J., Xie Y. (2021). Steel Fiber Reinforced Concrete: A Review of Its Material Properties and Usage in Tunnel Lining. Structures.

[13] Yang S., Wu Y., Zhou Z. (2024). Study on the Characteristics of Circumferential and Longitudinal Flow of Vault Concrete during Tunnel Lining Pouring Processes. Materials.

[14] Shang J.J.S. (2019). Landmarks and New Frontiers of Computational Fluid Dynamics. Adv. Aerodyn..

[15] Beccati N., Ferrari C., Bonanno A., Balestra M. (2019). Calibration of a CFD Discharge Process Model of an Off-Road Self-Loading Concrete Mixer. J. Braz. Soc. Mech. Sci. Eng..

[16] Wei J. (2019). Design and Application Study on Casting Concrete System with Layered and Window-by-window to Secondary Lining Side Wall in Tunnel. Railw. Constr. Technol..

[17] Zhang M., Xiao G., Wang S., Jia D., Gao C. (2020). Development of a New Type of Truss Type Large Clearance Lining Trolley for Railway Tunnel. J. Railw. Eng. Soc..

